# 1,2-Bis(2-methyl-5-phenyl-3-thien­yl)benzene

**DOI:** 10.1107/S1600536809045917

**Published:** 2009-11-07

**Authors:** Wen-Juan Miao, Liang-Hua Li, Shan Lu, Gang Liu, Cong-Bin Fan

**Affiliations:** a Jiangxi Key Laboratory of Organic Chemistry, Jiangxi Science & Technology Normal University, Nanchang 330013, People’s Republic of China; b East China Jiaotong University, School of Physical Education, Nanchang 330013, People’s Republic of China; c Department of Control Science and Engineering, Zhejiang University Zhejiang 310027, People’s Republic of China

## Abstract

In the mol­ecule of the title compound, C_28_H_22_S_2_, the two thio­phene rings are twisted with respect to the central benzene ring, making dihedral angles of 71.59 (12) and 50.71 (12)°. The two terminal benzene rings are oriented at dihedral angles of 37.59 (11) and 20.12 (11)° to their bonded thio­phene rings.

## Related literature

For the synthesis of the precursor, see: Irie *et al.* (2000 ▶). For applications of photochromic mol­ecules, see: Irie *et al.* (2001 ▶). For diaryl­ethenes with four different bridging units, see: Peters *et al.* (2003 ▶); Yamaguchi *et al.* (1997 ▶); Lucas *et al.* (1998 ▶); Chen & Zeng (2004 ▶). For ring-closure reactions, see: Ramamurthy & Venkatesan (1987 ▶). For a related structure, see: Pu *et al.* (2005 ▶).
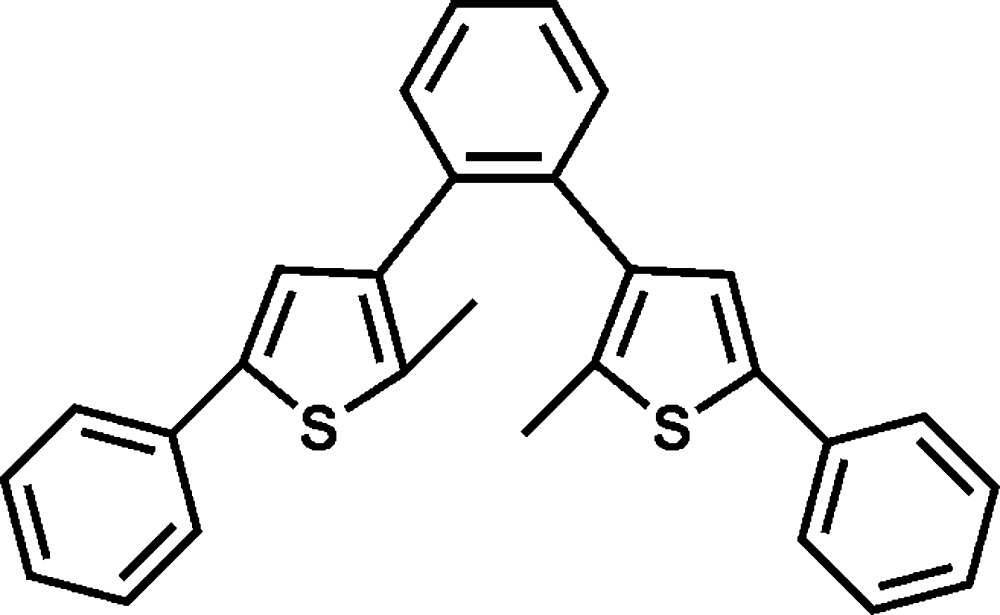


## Experimental

### 

#### Crystal data

C_28_H_22_S_2_
*M* *_r_* = 422.58
Triclinic, 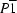

*a* = 10.0934 (12) Å
*b* = 10.0945 (12) Å
*c* = 11.9565 (15) Å
α = 83.803 (1)°
β = 67.769 (1)°
γ = 86.362 (1)°
*V* = 1120.7 (2) Å^3^
*Z* = 2
Mo *K*α radiation
μ = 0.25 mm^−1^
*T* = 296 K
0.40 × 0.24 × 0.15 mm

#### Data collection

Bruker SMART CCD area-detector diffractometer
Absorption correction: multi-scan (*SADABS*; Sheldrick, 1996 ▶) *T*_min_ = 0.907, *T*_max_ = 0.964
8610 measured reflections
4136 independent reflections
2920 reflections with *I* > 2σ(*I*)
*R*_int_ = 0.021

#### Refinement

*R*[*F* ^2^ > 2σ(*F* ^2^)] = 0.041
*wR*(*F* ^2^) = 0.108
*S* = 1.02
4136 reflections
273 parameters
H-atom parameters constrained
Δρ_max_ = 0.18 e Å^−3^
Δρ_min_ = −0.18 e Å^−3^

### 

Data collection: *SMART* (Bruker, 1997 ▶); cell refinement: *SAINT* (Bruker, 1997 ▶); data reduction: *SAINT*; program(s) used to solve structure: *SHELXTL* (Sheldrick, 2008 ▶); program(s) used to refine structure: *SHELXTL*; molecular graphics: *SHELXTL* software used to prepare material for publication: *SHELXTL*.

## Supplementary Material

Crystal structure: contains datablocks I, global. DOI: 10.1107/S1600536809045917/xu2660sup1.cif

Structure factors: contains datablocks I. DOI: 10.1107/S1600536809045917/xu2660Isup2.hkl

Additional supplementary materials:  crystallographic information; 3D view; checkCIF report

## Figures and Tables

**Fig. 1.**
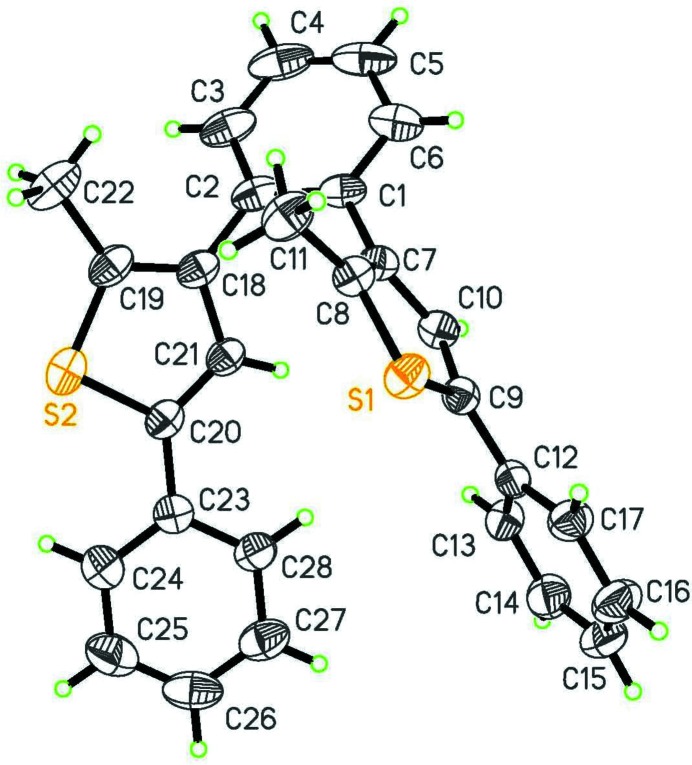
Molecular view the atom-labeling scheme. Ellipsoids are drawn at the 30% probability level. H atoms are represented as small spheres of arbitrary radii.

**Fig. 2.**
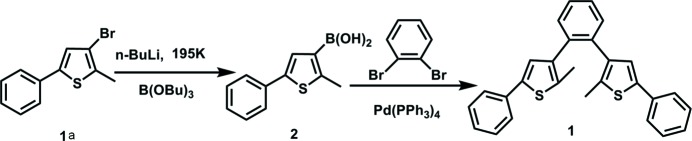
The preparation of the title compound.

## supplementary crystallographic information

### Comment

The design and synthesis of photochromic molecules is an area of intense research because of the widespread use in photonic device applications such as memory media and optical switching (Irie *et al.* 2001). To date, four kinds of diarylethenes with different bridge units have been reported, that is diarylethenes with a perfluorocyclopentene moiety (Peters *et al.* 2003), diarylethenes with maleic anhydride and maleimide moieties (Yamaguchi *et al.* 1997), diarylethenes with a cyclopentene moiety (Lucas *et al.* 1998), and diarylethenes with a 2,5-dihydrothiphene moiety (Chen & Zeng, 2004). One of our research goals is to develop a novel diarylethene derivative with the inexpensive benzene ring as bridge unit. In this paper, the *ORTEP* drawing of the single-crystal shows the title compound, *i.e.* 1,2-(2-methyl-5-phenyl-3-thienyl)benzene, packed in a parallel conformation which is very rare in other diarylethene system. The two independent planar thiophene ring systems have essentially identical geometries, and the dihedral angles between the central benzene-ring and these of the two thiophene rings, S1/C7—C10, and S2/ C18—C21 are 71.6 (4)° and 50.5 (7)°. The two thiophene groups are linked by the central benzene-ring, with both of them attached to the ethylene group *via* the 2-position. The distance between the two C atoms (C8···C19) is 4.06 (7) Å, which is short enough, theoretically, for the ring-closure reaction to take place in the crystalline phase (Ramamurthy & Venkatesan, 1987), but the crystals of the title compound is parallel thiophene-ring, so, the crystals cannot show photochromism.

### Experimental

2-Methyl-5-phenylthiophen-3-yl-3-boronic acid, (2) in Fig 2, was obtained in the presence of compound 3-bromo-2-methyl-5-phenylthiophene, (1), (Irie *et al.*, 2000) (2.53 g, 10.00 mmol) in a n-BuLi/hexane solution (2.50 mol/*L*, 12.00 mmol) and tri-n-butylborate (2.76 g, 12.00 mmol) at 195 K under a nitrogen atmosphere.

The title compound was prepared by adding compound (2) (0.88 g, 4.05 mmol) with Na_2_CO_3_ (2.00 mol/*L*, 60.00 mmol) to a stirred THF solution (50 ml) containing 1,2-dibromobenzene (0.48 g, 2.03 mmol) and Pd(PPh_3_)_4_ (0.27 g) at 293 K under a nitrogen atmosphere. After reflux for 16 h, the reaction mixture was extracted with ether, evaporated *in vacuo* and purified by column chromatography on SiO_2_ using petroleum ether as the eluent to obtain the title compound. Single crystals of the title compound (1a) were grown from a chloroform solution by slow evaporation (m.p. 404.8–405.2 K).

### Refinement

H atoms were placed in calculated positions and treated as riding with C—H = 0.96 Å (methyl) or 0.93 Å (aromatic), *U*_iso_(H) = 1.5*U*_eq_(C) for methyl H atoms and *U*_iso_(H) = 1.2*U*_eq_(C) for the others.

### Crystal data

**Table:**

C_28_H_22_S_2_	*Z* = 2
*M**_r_* = 422.58	*F*(000) = 444
Triclinic, *P*1	*D*_x_ = 1.252 Mg m^−^^3^
Hall symbol: -P 1	Mo *K*α radiation, λ = 0.71073 Å
*a* = 10.0934 (12) Å	Cell parameters from 2224 reflections
*b* = 10.0945 (12) Å	θ = 2.6–22.8°
*c* = 11.9565 (15) Å	µ = 0.25 mm^−^^1^
α = 83.803 (1)°	*T* = 296 K
β = 67.769 (1)°	Block, colourless
γ = 86.362 (1)°	0.40 × 0.24 × 0.15 mm
*V* = 1120.7 (2) Å^3^	

### Data collection

**Table:**

Bruker SMART CCD area-detector diffractometer	4136 independent reflections
Radiation source: fine-focus sealed tube	2920 reflections with *I* > 2σ(*I*)
graphite	*R*_int_ = 0.021
φ and ω scans	θ_max_ = 25.5°, θ_min_ = 2.3°
Absorption correction: multi-scan (*SADABS*; Sheldrick, 1996)	*h* = −12→12
*T*_min_ = 0.907, *T*_max_ = 0.964	*k* = −12→12
8610 measured reflections	*l* = −14→14

### Refinement

**Table:**

Refinement on *F*^2^	Primary atom site location: structure-invariant direct methods
Least-squares matrix: full	Secondary atom site location: difference Fourier map
*R*[*F*^2^ > 2σ(*F*^2^)] = 0.041	Hydrogen site location: inferred from neighbouring sites
*wR*(*F*^2^) = 0.108	H-atom parameters constrained
*S* = 1.02	*w* = 1/[σ^2^(*F*_o_^2^) + (0.0442*P*)^2^ + 0.2296*P*] where *P* = (*F*_o_^2^ + 2*F*_c_^2^)/3
4136 reflections	(Δ/σ)_max_ = 0.006
273 parameters	Δρ_max_ = 0.18 e Å^−^^3^
0 restraints	Δρ_min_ = −0.17 e Å^−^^3^

### Special details

**Table:**

Experimental. ^1^HNMR (400 MHz, CDCl_3_, TMS): δ 2.17 (s, 3H, -CH_3_), 6.92 (s, 1H, thienyl-H), 7.20 (t, 1H, *J*=7.2 Hz,phenyl-H), 7.29 (t, 2H, *J*=7.4 Hz, phenyl-H), 7.42–7.44 (m, 4H, phenyl-H). ^13^C NMR (100 MHz, CDCl_3_): δ 13.93, 125.51, 126.00, 127.00, 127.26, 128.76, 130.61, 134.60, 135.03, 136.09, 139.01, 139.45. IR (KBr, cm^-1^): 756, 766, 849, 908, 946, 1001, 1029, 1073, 1153, 1184, 1227, 1377, 1462, 1479, 1505, 1596, 1629, 2854, 2924, 3014, 3053, 3254, 3443, 3529, 3676.
Geometry. All e.s.d.'s (except the e.s.d. in the dihedral angle between two l.s. planes) are estimated using the full covariance matrix. The cell e.s.d.'s are taken into account individually in the estimation of e.s.d.'s in distances, angles and torsion angles; correlations between e.s.d.'s in cell parameters are only used when they are defined by crystal symmetry. An approximate (isotropic) treatment of cell e.s.d.'s is used for estimating e.s.d.'s involving l.s. planes.
Refinement. Refinement of *F*^2^ against ALL reflections. The weighted *R*-factor *wR* and goodness of fit *S* are based on *F*^2^, conventional *R*-factors *R* are based on *F*, with *F* set to zero for negative *F*^2^. The threshold expression of *F*^2^ > σ(*F*^2^) is used only for calculating *R*-factors(gt) *etc*. and is not relevant to the choice of reflections for refinement. *R*-factors based on *F*^2^ are statistically about twice as large as those based on *F*, and *R*- factors based on ALL data will be even larger.

### Fractional atomic coordinates and isotropic or equivalent isotropic displacement parameters (Å^2^)

**Table:**

	*x*	*y*	*z*	*U*_iso_*/*U*_eq_	
C1	0.8690 (3)	0.1761 (2)	0.33341 (19)	0.0593 (6)	
C2	1.0178 (3)	0.1571 (2)	0.27841 (19)	0.0632 (6)	
C3	1.0724 (3)	0.0279 (3)	0.2524 (2)	0.0837 (8)	
H3	1.1708	0.0137	0.2160	0.100*	
C4	0.9833 (5)	−0.0781 (3)	0.2798 (3)	0.1002 (11)	
H4	1.0217	−0.1633	0.2634	0.120*	
C5	0.8382 (5)	−0.0587 (3)	0.3312 (3)	0.0994 (11)	
H5	0.7780	−0.1303	0.3478	0.119*	
C6	0.7806 (3)	0.0679 (2)	0.3587 (2)	0.0803 (7)	
H6	0.6819	0.0803	0.3944	0.096*	
C7	0.8057 (2)	0.3112 (2)	0.36380 (18)	0.0534 (5)	
C8	0.8183 (2)	0.3757 (2)	0.45372 (19)	0.0564 (5)	
C9	0.6907 (2)	0.5132 (2)	0.33745 (18)	0.0517 (5)	
C10	0.7318 (2)	0.3902 (2)	0.29899 (19)	0.0563 (5)	
H10	0.7133	0.3602	0.2357	0.068*	
C11	0.8878 (3)	0.3254 (3)	0.5415 (2)	0.0791 (7)	
H11A	0.9079	0.2315	0.5370	0.119*	
H11B	0.8245	0.3414	0.6223	0.119*	
H11C	0.9755	0.3712	0.5214	0.119*	
C12	0.6259 (2)	0.6253 (2)	0.28537 (19)	0.0535 (5)	
C13	0.6640 (2)	0.6476 (2)	0.1601 (2)	0.0626 (6)	
H13	0.7277	0.5890	0.1095	0.075*	
C14	0.6079 (3)	0.7558 (3)	0.1111 (3)	0.0798 (7)	
H14	0.6339	0.7703	0.0274	0.096*	
C15	0.5132 (3)	0.8426 (3)	0.1857 (3)	0.0821 (8)	
H15	0.4765	0.9163	0.1523	0.099*	
C16	0.4735 (3)	0.8211 (3)	0.3071 (3)	0.0855 (8)	
H16	0.4077	0.8791	0.3568	0.103*	
C17	0.5293 (2)	0.7141 (2)	0.3585 (2)	0.0692 (6)	
H17	0.5022	0.7013	0.4424	0.083*	
C18	1.1146 (2)	0.2710 (2)	0.24104 (19)	0.0586 (6)	
C19	1.2360 (3)	0.2804 (2)	0.2660 (2)	0.0659 (6)	
C20	1.1898 (2)	0.4834 (2)	0.14581 (18)	0.0552 (5)	
C21	1.0915 (2)	0.3879 (2)	0.17170 (18)	0.0554 (5)	
H21	1.0148	0.3978	0.1462	0.066*	
C22	1.2975 (3)	0.1831 (3)	0.3393 (3)	0.0933 (9)	
H22A	1.2255	0.1213	0.3891	0.140*	
H22B	1.3286	0.2306	0.3899	0.140*	
H22C	1.3775	0.1354	0.2855	0.140*	
C23	1.1947 (2)	0.6143 (2)	0.07776 (19)	0.0567 (5)	
C24	1.3183 (3)	0.6851 (3)	0.0253 (3)	0.0844 (8)	
H24	1.4020	0.6492	0.0327	0.101*	
C25	1.3216 (4)	0.8071 (3)	−0.0376 (3)	0.1023 (10)	
H25	1.4068	0.8529	−0.0719	0.123*	
C26	1.2003 (4)	0.8618 (3)	−0.0504 (2)	0.0908 (9)	
H26	1.2025	0.9452	−0.0926	0.109*	
C27	1.0761 (4)	0.7937 (3)	−0.0010 (2)	0.0833 (8)	
H27	0.9936	0.8298	−0.0108	0.100*	
C28	1.0726 (3)	0.6706 (2)	0.0638 (2)	0.0664 (6)	
H28	0.9871	0.6252	0.0984	0.080*	
S1	0.74075 (6)	0.53246 (6)	0.45767 (5)	0.06329 (19)	
S2	1.31728 (6)	0.43038 (7)	0.20603 (6)	0.0720 (2)	

### Atomic displacement parameters (Å^2^)

**Table:**

	*U*^11^	*U*^22^	*U*^33^	*U*^12^	*U*^13^	*U*^23^
C1	0.0884 (17)	0.0445 (12)	0.0466 (12)	−0.0012 (11)	−0.0277 (12)	−0.0017 (9)
C2	0.0946 (18)	0.0483 (13)	0.0484 (12)	0.0156 (12)	−0.0301 (12)	−0.0083 (10)
C3	0.131 (2)	0.0569 (16)	0.0672 (16)	0.0299 (16)	−0.0444 (16)	−0.0162 (13)
C4	0.188 (4)	0.0461 (16)	0.081 (2)	0.021 (2)	−0.068 (2)	−0.0143 (14)
C5	0.184 (4)	0.0475 (16)	0.080 (2)	−0.023 (2)	−0.066 (2)	0.0057 (14)
C6	0.118 (2)	0.0591 (16)	0.0649 (16)	−0.0183 (15)	−0.0345 (15)	0.0021 (12)
C7	0.0589 (13)	0.0478 (12)	0.0491 (12)	−0.0035 (10)	−0.0159 (10)	−0.0009 (9)
C8	0.0610 (13)	0.0558 (13)	0.0527 (12)	0.0030 (10)	−0.0217 (10)	−0.0075 (10)
C9	0.0501 (11)	0.0513 (12)	0.0501 (12)	−0.0015 (9)	−0.0141 (9)	−0.0067 (10)
C10	0.0634 (13)	0.0543 (13)	0.0525 (12)	−0.0050 (10)	−0.0219 (11)	−0.0085 (10)
C11	0.0984 (19)	0.0797 (17)	0.0720 (16)	0.0163 (15)	−0.0466 (15)	−0.0164 (13)
C12	0.0456 (11)	0.0528 (12)	0.0599 (13)	−0.0046 (9)	−0.0166 (10)	−0.0054 (10)
C13	0.0586 (13)	0.0684 (15)	0.0603 (14)	0.0055 (11)	−0.0224 (11)	−0.0072 (11)
C14	0.0778 (17)	0.0892 (19)	0.0730 (17)	−0.0020 (15)	−0.0326 (14)	0.0068 (15)
C15	0.0871 (19)	0.0654 (16)	0.090 (2)	0.0093 (14)	−0.0358 (16)	0.0098 (15)
C16	0.0881 (19)	0.0652 (17)	0.091 (2)	0.0222 (14)	−0.0230 (16)	−0.0115 (15)
C17	0.0703 (15)	0.0608 (15)	0.0668 (15)	0.0054 (12)	−0.0164 (12)	−0.0032 (12)
C18	0.0679 (14)	0.0547 (13)	0.0486 (12)	0.0163 (11)	−0.0177 (11)	−0.0113 (10)
C19	0.0751 (15)	0.0671 (15)	0.0543 (13)	0.0249 (12)	−0.0247 (12)	−0.0144 (11)
C20	0.0573 (13)	0.0598 (13)	0.0472 (12)	0.0106 (11)	−0.0181 (10)	−0.0116 (10)
C21	0.0596 (13)	0.0561 (13)	0.0490 (12)	0.0106 (11)	−0.0195 (10)	−0.0081 (10)
C22	0.115 (2)	0.0891 (19)	0.0877 (19)	0.0411 (17)	−0.0562 (18)	−0.0157 (15)
C23	0.0633 (14)	0.0582 (13)	0.0478 (12)	0.0019 (11)	−0.0185 (11)	−0.0124 (10)
C24	0.0715 (17)	0.0837 (19)	0.092 (2)	−0.0104 (14)	−0.0261 (15)	0.0034 (16)
C25	0.107 (2)	0.086 (2)	0.100 (2)	−0.0264 (19)	−0.025 (2)	0.0139 (18)
C26	0.149 (3)	0.0630 (17)	0.0606 (16)	−0.011 (2)	−0.0398 (19)	0.0025 (13)
C27	0.114 (2)	0.0696 (17)	0.0803 (18)	0.0118 (17)	−0.0540 (18)	−0.0101 (14)
C28	0.0756 (16)	0.0585 (14)	0.0708 (15)	0.0037 (12)	−0.0347 (13)	−0.0059 (12)
S1	0.0722 (4)	0.0584 (4)	0.0657 (4)	0.0089 (3)	−0.0312 (3)	−0.0185 (3)
S2	0.0651 (4)	0.0846 (5)	0.0711 (4)	0.0146 (3)	−0.0318 (3)	−0.0135 (3)

### Geometric parameters (Å, °)

**Table:**

C1—C6	1.389 (3)	C14—H14	0.9300
C1—C2	1.403 (3)	C15—C16	1.348 (4)
C1—C7	1.490 (3)	C15—H15	0.9300
C2—C3	1.403 (3)	C16—C17	1.380 (3)
C2—C18	1.475 (3)	C16—H16	0.9300
C3—C4	1.373 (4)	C17—H17	0.9300
C3—H3	0.9300	C18—C19	1.377 (3)
C4—C5	1.367 (4)	C18—C21	1.427 (3)
C4—H4	0.9300	C19—C22	1.505 (3)
C5—C6	1.390 (4)	C19—S2	1.720 (3)
C5—H5	0.9300	C20—C21	1.354 (3)
C6—H6	0.9300	C20—C23	1.468 (3)
C7—C8	1.364 (3)	C20—S2	1.730 (2)
C7—C10	1.422 (3)	C21—H21	0.9300
C8—C11	1.500 (3)	C22—H22A	0.9600
C8—S1	1.718 (2)	C22—H22B	0.9600
C9—C10	1.356 (3)	C22—H22C	0.9600
C9—C12	1.469 (3)	C23—C24	1.373 (3)
C9—S1	1.727 (2)	C23—C28	1.384 (3)
C10—H10	0.9300	C24—C25	1.367 (4)
C11—H11A	0.9600	C24—H24	0.9300
C11—H11B	0.9600	C25—C26	1.366 (4)
C11—H11C	0.9600	C25—H25	0.9300
C12—C17	1.390 (3)	C26—C27	1.362 (4)
C12—C13	1.394 (3)	C26—H26	0.9300
C13—C14	1.375 (3)	C27—C28	1.388 (3)
C13—H13	0.9300	C27—H27	0.9300
C14—C15	1.376 (4)	C28—H28	0.9300
			
C6—C1—C2	119.4 (2)	C16—C15—H15	119.9
C6—C1—C7	120.1 (2)	C14—C15—H15	119.9
C2—C1—C7	120.50 (19)	C15—C16—C17	120.8 (2)
C1—C2—C3	118.4 (2)	C15—C16—H16	119.6
C1—C2—C18	121.09 (18)	C17—C16—H16	119.6
C3—C2—C18	120.4 (2)	C16—C17—C12	120.1 (2)
C4—C3—C2	121.2 (3)	C16—C17—H17	119.9
C4—C3—H3	119.4	C12—C17—H17	119.9
C2—C3—H3	119.4	C19—C18—C21	111.2 (2)
C5—C4—C3	120.2 (3)	C19—C18—C2	126.3 (2)
C5—C4—H4	119.9	C21—C18—C2	122.4 (2)
C3—C4—H4	119.9	C18—C19—C22	129.2 (2)
C4—C5—C6	120.1 (3)	C18—C19—S2	111.29 (17)
C4—C5—H5	120.0	C22—C19—S2	119.5 (2)
C6—C5—H5	120.0	C21—C20—C23	127.9 (2)
C1—C6—C5	120.7 (3)	C21—C20—S2	109.85 (16)
C1—C6—H6	119.7	C23—C20—S2	122.28 (18)
C5—C6—H6	119.7	C20—C21—C18	114.9 (2)
C8—C7—C10	112.25 (19)	C20—C21—H21	122.5
C8—C7—C1	123.57 (19)	C18—C21—H21	122.5
C10—C7—C1	124.11 (19)	C19—C22—H22A	109.5
C7—C8—C11	128.1 (2)	C19—C22—H22B	109.5
C7—C8—S1	110.85 (16)	H22A—C22—H22B	109.5
C11—C8—S1	121.04 (16)	C19—C22—H22C	109.5
C10—C9—C12	129.5 (2)	H22A—C22—H22C	109.5
C10—C9—S1	109.77 (16)	H22B—C22—H22C	109.5
C12—C9—S1	120.54 (15)	C24—C23—C28	117.4 (2)
C9—C10—C7	114.25 (19)	C24—C23—C20	122.3 (2)
C9—C10—H10	122.9	C28—C23—C20	120.3 (2)
C7—C10—H10	122.9	C25—C24—C23	121.8 (3)
C8—C11—H11A	109.5	C25—C24—H24	119.1
C8—C11—H11B	109.5	C23—C24—H24	119.1
H11A—C11—H11B	109.5	C26—C25—C24	120.3 (3)
C8—C11—H11C	109.5	C26—C25—H25	119.9
H11A—C11—H11C	109.5	C24—C25—H25	119.9
H11B—C11—H11C	109.5	C27—C26—C25	119.6 (3)
C17—C12—C13	118.3 (2)	C27—C26—H26	120.2
C17—C12—C9	121.3 (2)	C25—C26—H26	120.2
C13—C12—C9	120.39 (19)	C26—C27—C28	120.0 (3)
C14—C13—C12	120.4 (2)	C26—C27—H27	120.0
C14—C13—H13	119.8	C28—C27—H27	120.0
C12—C13—H13	119.8	C23—C28—C27	120.9 (2)
C13—C14—C15	120.0 (2)	C23—C28—H28	119.6
C13—C14—H14	120.0	C27—C28—H28	119.6
C15—C14—H14	120.0	C8—S1—C9	92.87 (10)
C16—C15—C14	120.3 (2)	C19—S2—C20	92.73 (11)
			
C6—C1—C2—C3	−1.0 (3)	C9—C12—C17—C16	177.9 (2)
C7—C1—C2—C3	179.48 (19)	C1—C2—C18—C19	131.3 (2)
C6—C1—C2—C18	175.0 (2)	C3—C2—C18—C19	−52.8 (3)
C7—C1—C2—C18	−4.6 (3)	C1—C2—C18—C21	−48.9 (3)
C1—C2—C3—C4	0.0 (3)	C3—C2—C18—C21	126.9 (2)
C18—C2—C3—C4	−176.0 (2)	C21—C18—C19—C22	177.8 (2)
C2—C3—C4—C5	1.3 (4)	C2—C18—C19—C22	−2.4 (4)
C3—C4—C5—C6	−1.6 (4)	C21—C18—C19—S2	0.5 (2)
C2—C1—C6—C5	0.7 (3)	C2—C18—C19—S2	−179.78 (16)
C7—C1—C6—C5	−179.8 (2)	C23—C20—C21—C18	−179.37 (19)
C4—C5—C6—C1	0.6 (4)	S2—C20—C21—C18	0.6 (2)
C6—C1—C7—C8	110.3 (3)	C19—C18—C21—C20	−0.7 (3)
C2—C1—C7—C8	−70.1 (3)	C2—C18—C21—C20	179.54 (18)
C6—C1—C7—C10	−73.0 (3)	C21—C20—C23—C24	−159.5 (2)
C2—C1—C7—C10	106.6 (2)	S2—C20—C23—C24	20.6 (3)
C10—C7—C8—C11	179.6 (2)	C21—C20—C23—C28	20.2 (3)
C1—C7—C8—C11	−3.3 (4)	S2—C20—C23—C28	−159.79 (17)
C10—C7—C8—S1	−0.4 (2)	C28—C23—C24—C25	0.5 (4)
C1—C7—C8—S1	176.70 (17)	C20—C23—C24—C25	−179.8 (3)
C12—C9—C10—C7	173.27 (19)	C23—C24—C25—C26	−0.3 (5)
S1—C9—C10—C7	−1.0 (2)	C24—C25—C26—C27	−0.6 (5)
C8—C7—C10—C9	0.9 (3)	C25—C26—C27—C28	1.2 (4)
C1—C7—C10—C9	−176.1 (2)	C24—C23—C28—C27	0.1 (3)
C10—C9—C12—C17	147.8 (2)	C20—C23—C28—C27	−179.5 (2)
S1—C9—C12—C17	−38.5 (3)	C26—C27—C28—C23	−1.0 (4)
C10—C9—C12—C13	−34.4 (3)	C7—C8—S1—C9	−0.15 (17)
S1—C9—C12—C13	139.39 (18)	C11—C8—S1—C9	179.88 (19)
C17—C12—C13—C14	0.7 (3)	C10—C9—S1—C8	0.66 (17)
C9—C12—C13—C14	−177.3 (2)	C12—C9—S1—C8	−174.21 (17)
C12—C13—C14—C15	−0.2 (4)	C18—C19—S2—C20	−0.12 (17)
C13—C14—C15—C16	−0.9 (4)	C22—C19—S2—C20	−177.74 (19)
C14—C15—C16—C17	1.6 (4)	C21—C20—S2—C19	−0.27 (16)
C15—C16—C17—C12	−1.1 (4)	C23—C20—S2—C19	179.69 (17)
C13—C12—C17—C16	0.0 (3)		
